# Change in auditory evoked potential index and bispectral index during induction of anesthesia with anesthetic drugs

**DOI:** 10.1007/s10877-014-9643-x

**Published:** 2014-11-27

**Authors:** Sachiko Matsushita, Shinya Oda, Kei Otaki, Masaki Nakane, Kaneyuki Kawamae

**Affiliations:** Department of Anesthesiology, Faculty of Medicine, Yamagata University, Iida-nishi 2-2-2, Yamagata-shi, Yamagata, 990-2323 Japan

**Keywords:** Auditory evoked potential, Bispectral index, Depth of anesthesia monitors

## Abstract

The aim of this study was to evaluate the efficacy of the auditory evoked potential (AEP) index (aepEX) as an assessment tool for hypnosis during induction of various anesthetic drugs, and to compare its performance to that of the bispectral index (BIS). A total of 45 cases were divided into three groups based on the drugs used for anesthesia. Before anesthetic induction, BIS and AEP monitors were initiated. Anesthesia was induced through intravenous injection (IV) as follows: MP (n = 15) group, midazolam (0.1 mg/kg IV); TP (n = 15) group, thiopental (4 mg/kg IV); and KP (n = 15) group, ketamine (2 mg/kg IV). After loss of response (LOR), an infusion of 3 μg/ml propofol via a target-controlled infusion was initiated in all groups. AepEX and BIS were measured in the waking state (baseline) and at LOR (1 min after LOR), pre-intubation (1 min after previous intubation) and post-intubation (1 min after tracheal intubation finished). The value of aepEX significantly decreased in all groups with LOR and that of BIS also decreased except of KP group. No significant difference were observed in BIS values between baseline and LOR in the KS group. The aepEX might be more useful than BIS for hypnosis monitoring during anesthetic induction, particularly when drugs such as ketamine are used.

## Introduction

Many studies have used electroencephalographic (EEG) parameters to estimate the depth of anesthesia and sedation [1–4]. Nowadays, bispectral index (BIS), which is generated through computation of spontaneous EEG information, is the most popular EEG-based monitoring index for hypnotic depth assessment [5].

Auditory evoked potentials (AEP) are the electrical potentials evoked in the auditory pathway in response to sound stimuli, measurement of which has been proposed as a reliable method for the assessment of anesthetic depth during general anesthesia [2]. It has been reported that the middle latency components in the AEP well reflect the degree of central nervous depression in response to anesthetics [6, 7].

The aepEXplus™, Medical Device Management, Essex, UK, a new AEP monitor for measuring the AEP index (aepEX), has recently become available. The aepEXplus primarily analyzes middle latency auditory evoked potential (MLAEP), which is susceptible to the effect of sedatives, and uses it to calculate the aepEX. The aepEX is derived by summing the square root of the absolute difference between successive 0.56-ms epochs of the AEP waveforms (up to 144 ms from sound stimulus) [2]. It has been observed that when patients lose consciousness after multiple anesthetic administrations, AEP peak amplitudes decrease as their latencies increase, which leads to a decrease in aepEX at loss of consciousness [6, 7]. Judging from these facts, aepEX may be the index that is useful as indicator of the hypnosis effect of most anesthetics.

The present study was performed to assess the changes in aepEX with three anesthetic induction agents, thiopental, midazolam and ketamine, in combination with propofol, and to compare these changes with those in BIS values at the same time points.

### Objectives

The aim of this study was to investigate the efficacy of the aepEX as a measure of sedative effect during anesthetic induction with different drug groups. To serve as a standard, BIS was measured and compared with aepEX concurrently.

## Methods

### Patients and study design

Approval was taken from institutional ethical review committee and written informed consent was taken from each patient. We studied 45 patients (ASA physical status between I and II; age 22–79 years) who received general anesthesia without premedication for elective surgery. The types of surgeries performed were thoracic, abdominal, urological, orthopedic and dermatological surgery. Exclusion criteria included severe cardiac, respiratory, liver, kidney and neurological dysfunction, and auditory impairment. We did not include a patient with hearing loss, it was not performed the preoperative hearing test in all cases. Patients were also excluded if they were receiving medication that could potentially affect cerebral function, such as sedatives, antidepressants and anticonvulsants. Forty-five patients were allocated to three study groups based on the drugs used for anesthetic induction as shown in Table 1, using sealed envelopes as a randomization. In all patients, aepEX and BIS were measured from the time of induction to post-intubation.

**Table 1:** Patient characteristics

	MP group (n=15)	MP group (n=15)	MP group (n=15)	*P* values
Age (years)	54.1 ± 7.1	55.8 ± 4.0	56.8 ± 3.6	0.924
Gender (male/female)	6/5	6/8	9/6	0.284
Weight (kg)	55.8 ± 4.8	61.5 ± 13.8	59.9 ± 11.2	0.455
Height (cm)	161.9 ± 8.7	159.9 ± 8.7	161.5 ± 9.2	0.823
BMI	21.4 ± 2.4	24.2 ± 3.9	22.9 ± 3.3	0.140
ASA stage I/II (n)	2/13	5/10	7/8	0.284

Data are expressed as mean ± SD

ASA american society of anesthesiologists

### Anesthesia

**Fig. 1:**
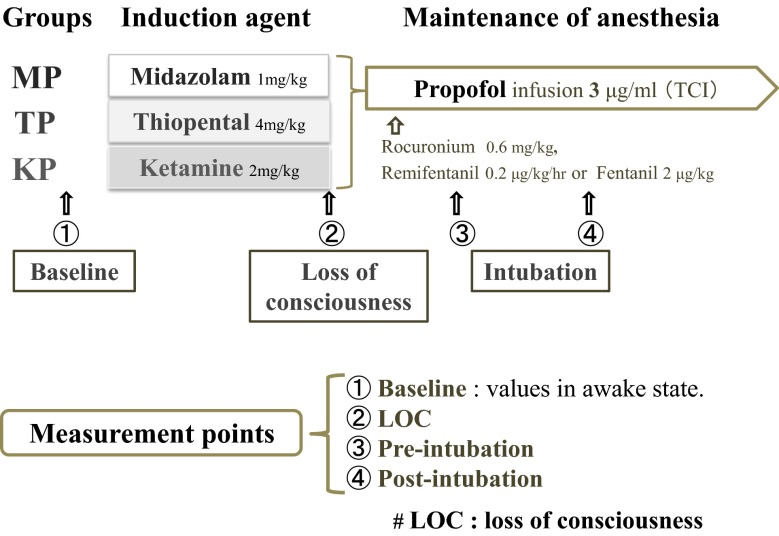
Experimental protocol

Anesthesia was administered as shown in Fig. 1. Under inhalation of 5 l/min of oxygen via a face mask, anesthesia was induced with bolus administration of the anesthetics. The MP (n = 15), TP (n = 15) and KP groups (n = 15) were given midazolam (0.1 mg/kg IV), thiopental (4.0 mg/kg IV) and ketamine (2.0 mg/kg IV), respectively, for anesthetic induction, and an infusion of 3 μg/ml propofol via a target-controlled infusion after loss of responsiveness (LOR). LOR was determined by the loss of response to verbal commands and loss of the eyelash reflex. After LOR, all patients received a remifentanil (0.2 μg/kg/min) infusion or fentanyl (2 μg/kg IV) injection for analgesia, and a rocuronium (0.6 mg/kg IV) injection for muscle relaxation in order to assist endotracheal intubation. Measurements were performed at the following time points as shown in Fig. 1: (1) baseline values in the waking state; (2) at LOR (1 min after LOR); (3) at pre-intubation (1 min after previous intubation); and (4) at post-intubation (1 min after tracheal intubation finished).

### Monitoring

The monitors used in this study comprised the BIS monitor (BIS model A-2000™ XP, software version 2.21, Aspect Medical Systems, Boston, MA, USA) and the aepEX monitor (aepEXplus™, Covidien, Medical Device Management, Dublin, Ireland) [8, 9].

Monitoring of both aepEX and BIS was initiated before the induction of anesthesia. Baseline values of aepEX and BIS were measured after a 3–5 min period of rest in the supine position. Thereafter, aepEX and BIS data were continuously recorded during the study period, which was from the time before induction of anesthesia until after tracheal intubation. The BIS probe was placed on the forehead of each patient. BIS was calculated from the latest 61.5 s of EEG signals, using several parameters.

### AepEX

AepEX values were obtained using three electrodes, one placed on the middle of the forehead as a reference, the second placed nearby on the forehead and the third placed on the mastoid process on the same side as the second electrode. After the skin was prepared with alcohol and a gentle abrasive, electrodes specially supplied by the manufacturer were attached. Electrode impedances were considered acceptable if they were <5 kΩ. Click sounds were presented via earphones at a frequency of 6.9 Hz (one click every 144 ms) and intensity of 90 decibels above the normal hearing level. The AEPs averaged 256 sweeps of 144 ms duration. The time required for a full update of the signals was 36.9 s, and AEPs were obtained at intervals of 3 s with moving-average method [2]. AepEX was calculated by analysis of the AEP waveform. AepEX is derived by summing the square root of the absolute difference between successive amplitudes of the AEP waveforms (for 144 ms from presentation of the sound stimulus). AepEX mainly reflects the middle latency auditory evoked potential (MLAEP), which originates from the part of the auditory pathway from the medial geniculate body to the primary auditory cortex (acoustic radiation). When anesthetic agents are administered, resulting in LOR, the MLAEP peak amplitudes reduce and their latencies are extended. Hence, the value of aepEX decreases when patients lose responsiveness.

### Statistics

Calculation of sample size was performed using The PS—power and sample size Calculation program version 3.0.43, program by William D. Dupont and W. Dale Plummer, Jr. The number of patients per group was determined based on our preliminary experiments. We hypothesized that the value of aepEX in the LOR decrease by −20 % compared with that in the baseline. We determined that 10 members to provide Type I error protection of 0.05 and a power of 0.80 to detect a −20 % decrease in the value of aepEX, between at baseline and LOR. Data are expressed as the mean ± SD, or median ± quartile deviation. One way analysis of variance (ANOVA) and Chi squared test were used to assess the differences in patient characteristics between the groups. Since values of aepEX and BIS were not normally distributed, the measured values are expressed as median ± quartile deviation. A value of *P* < 0.05 was considered statistically significant. AepEX and BIS were compared among the different groups by Steel test. All data were statistically analyzed using a commercially available software package (JMP version 9.0, SAS Institute Inc. Cary, NC, USA).

## Results

The patients’ demographics are shown in Table 1. No significant differences were seen between groups in terms of age, sex, weight, height and BMI. ASA physical status of the patients in each group is shown in Table 1.

**Fig. 2:**
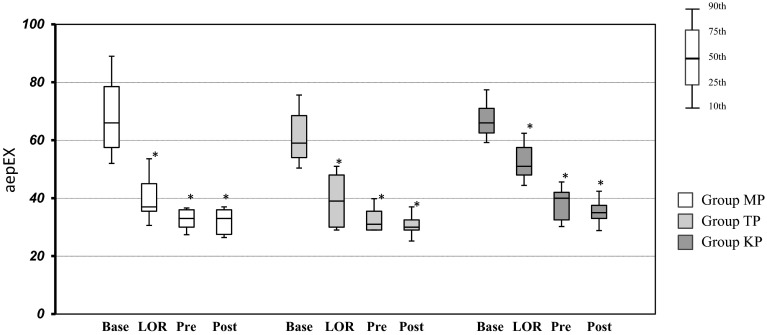
Comparison between aepEX values in three groups at the different measurement points. *Box plots* show median and 25th and 75th percentiles (box boudaries) and 10th and 90th percentiles (whiskers). *P < 0.01 versus baseline

**Fig. 3:**
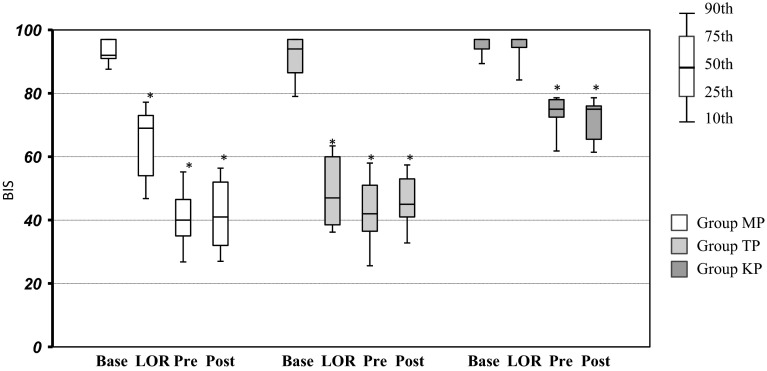
Comparison between BIS values in the six groups at the different measurement points. Box plots show median and 25th and 75th percentiles (box boudaries) and 10th and 90th percentiles (whiskers). *P < 0.01 versus baseline

Figure 2 shows the aepEX values in the three groups at the different measurement points. aepEX values showed a decline in all groups with LOR, and a gradual decrease thereafter to post-intubation levels, as seen in Fig. 2. Significant differences were observed in aepEX values in all groups between baseline and LOR. BIS values showed a similar decrease at LOR in the MP and TP groups, although the value of BIS at LOR in the KP groups was almost the same as their value at baseline. No significant differences were observed in BIS values in the KP groups between baseline and LOR (Fig. 3). Additionally, BIS values in the KP group both pre- and post-intubation, which were 75 (interquartile range, 72.2–77.8), 75 (interquartile range, 69.7–80.3) respectively, tended to be higher than those in the other groups at the same time points. BIS in the KP group shows a high value about 70 even in (pre-, post-intubation) at a point in time when it is thought that sedation is deep enough. Baseline aepEX values showed more variability than baseline BIS values.

## Discussion

In this study, aepEX values decreased significantly with LOR after anesthetic induction in all groups, mirroring the clinical symptoms. Conversely, although BIS values showed significant decreases with LOR in the MP and TP groups, similar to that seen in aepEX values, no significant decrease was observed in BIS values with LOR compared to baseline in the KP group. Thus, in the groups that received midazolam and thiopental for anesthetic induction, the decrease in BIS values coincided with clinical LOR. The group receiving ketamine, on the other hand, had higher BIS values despite LOR, showing dissociation with clinical symptoms in the ketamine-induced hypnotic state.

Evoked potentials induced by auditory stimuli are classified according to the interval between stimulus and response as short latency AEP (SLAEP), middle latency AEP (MLAEP) and long latency AEP (LLAEP). SLAEP is the potential generated immediately after presentation of the auditory stimulus, known as the auditory brainstem response (ABR), which originates from the brainstem. Since ABR is minimally affected by anesthetic agents, it plays an important role in the determination of brainstem function during anesthesia. MLAEP, which follows ABR, has a latency of about 10 ms and is generated from the primary auditory cortex to the medial geniculate body [7]. MLAEP has been studied as an indicator of the degree of sedation because the amplitude is reduced and the latency is extended in proportion to the concentration of anesthetic administered [2, 10]. The LLAEP originates in the association area of the cerebral cortex.

A key difference between BIS and aepEX is that aepEX is the numerical value acquired using MLAEP, which is an evoked potential. MLAEP is a weak current generated by excitement of the cranial nerves during information processing in the brain. In contrast, BIS is the numerical value acquired using the spontaneous electroencephalogram (EEG). It has been reported that aepEX is superior to BIS in determining the state of consciousness [10, 11]. It has also been reported that aepEX values have superior predictive power of the likelihood of patient movement in response to skin incision [2].

- BIS is a passive measurement method used to monitor the central nervous system during anesthesia, accompanied by spontaneous EEG analysis of the frontal cortex. BIS was calculated using several parameters (relative beta-ratio, synch-fast-slow, burst-suppression ratio, etc.), which were obtained from multiple methods of analysis. In addition, due to the fact that the quantification of BIS involves numerical adjustment weighted to the empirical EEG database [3, 4], it is an estimate as opposed to a pure measured value. Glass et al. [3] have described that the EEG database used to calculate BIS contains the EEG during administration of isoflurane, thiopental, propofol and midazolam with opioids and N_2_O. BIS has been shown to accurately represent the extent of sedation-hypnosis induced by various anesthetic agents [3]. However, BIS may not adequately reflect the degree of sedation when drugs that are not included in the database of the BIS calculation are administered; hence, anesthesiologists need to be cautious about the interpretation of BIS in these cases [12].

**Fig. 4:**
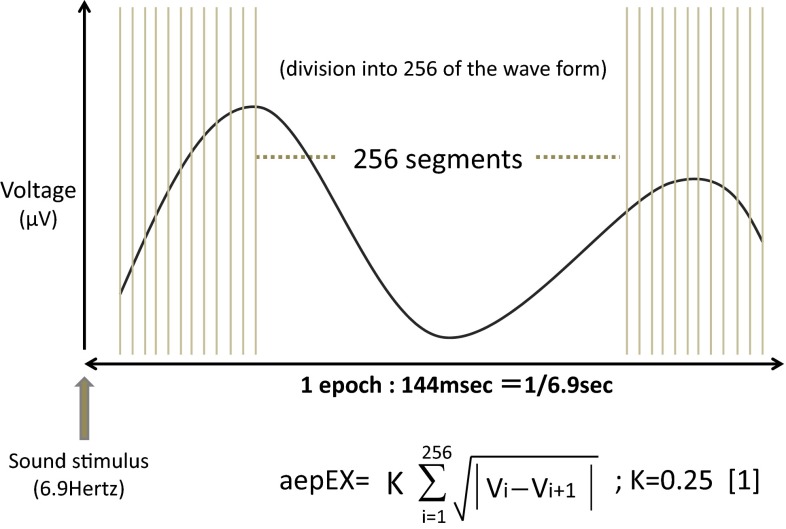
The calculation method of aepEX. One epoch (144 ms) divides into 256 pieces and the value of aepEX is acquired from a formula [1]

In this study, large variations were observed in the aepEX as compared to BIS values at baseline. This is because aepEX values are obtained by calculating the amplitude of auditory evoked potentials, and thus have individual differences. These trends in variability were relatively reversed during the period from LOR until after tracheal intubation, the trend being for less variation in aepEX. The value of aepEX, it is calculated based on the AEP waveform (up to 144 ms from auditory stimuli) as shown in Fig. 4, so it is considered to contain the waveform component ABR, N0, P0, Na, Pa, Nb such as P1 in awake state. MLAEP components Nb, P1 have large individual differences of amplitude, in some cases that waveforms are disappeared [13, 14]. Since it is derived from the waveform containing the MLAEP components Nb and P1, it is thought that the value of aepEX in awake state has large individual difference. As you deep sedation, latency of MLAEP to extend, so the value of aepEX has mainly reflected by MLAEP components of N0, P0, Na, and Pa which have relatively less individual variability than Nb such as P1. Therefore variability of aepEX value may become small as it sedation.

Administration of many anesthetics, such as barbiturates, benzodiazepines and propofol, is associated with a progressive change in the EEG from low amplitude fast waves to high amplitude slow waves, with increasing anesthetic concentration in the brain [15]. Reportedly, these intravenous anesthetics cause suppression of the central nervous system via action of the synaptic activating system on the GABAA receptor [15]. Ketamine, on the other hand, inhibits NMDA-mediated glutamatergic inputs to GABAergic interneurons, leading to aberrant excitatory activity in the cortex, hippocampus and limbic system, and ultimately unconsciousness [15]. Ketamine acts on the thalamocortical projection system and depresses neuronal function in parts of the cortex (especially association areas) and thalamus [16]. Therefore, ketamine exhibits different EEG changes than other anesthetics [15, 17]. The EEG pattern under ketamine anesthesia is characterized by frontally dominant rhythmic high-amplitude theta activity, with increasing doses of ketamine producing high-amplitude polymorphic delta activity, interspersed with low-amplitude beta activity [18]. It has been reported that BIS either tends to remain high in comparison with the degree of sedation or does not decrease with ketamine use, although clinically, patients are unconsciousness at the time [19, 20]. This is thought to be a result of the cerebral excitatory activity produced by ketamine. Due to this gap between actual clinical symptoms and EEG activity, evaluation of ketamine sedation by BIS is considered to be difficult.

There has been little research on the numerical changes in aepEX with ketamine administration. Reportedly, no change is observed in MLAEP with a 2 mg/kg induction dose of ketamine [21]. Therefore, it is speculated that the anesthetic effect of ketamine relies on a disturbed stimulus evaluation in the secondary associative cortex. Since aepEX is computed using the auditory evoked potential of sweeps of 144 ms duration, it might also contain some LLAEP. In contrast, the duration of waveform is 100 ms had measured in the study of Schwender et al. [21]. The segment of AEP between 100 and 144 ms corresponds to LLAEP. LLAEP comes from cortical association areas and is potential to express the sound recognition. Hence, in this study, for this difference between 100 and 144 ms, the value of aepEX might have been reduced with the use of ketamine, which depresses neural function in the cortical association area. In this study, aepEX showed a decrease at LOR, pre- and post-intubation with ketamine administration. On the other hand, the value of BIS with ketamine administration shows a high value about 70 even in (pre-, post-intubation) at a point in time when sedation is deep enough. This may suggest the possibility that aepEX is useful for the evaluation of the sedation state in the ketamine dosage.

This study has several limitations. It is difficult to completely eliminate ambient noise, such as that due to electromyograms and electric interference by various electronic devices in the operation room. Although we attempted to completely remove extraneous sound while measuring aepEX, some variables might have been affected by these artifacts.

In this study, we measured and estimated the index for bolus administration of the induction medicine that is clinically popular induction method. Since the bolus administration of induction drugs was carried out, the concentration of drug within a brain may be uneven. However since we measured the both of BIS and aepEX at the same time, it is possible to compare the both values in the same sedation state each other.

Thiopental is known to occur a huge delta wave at the level of not so deep sedation, there is a possibility of having overestimated the grade of hypnosis. Even if a sedation level can be overestimated in a value in BIS, it is less likely to be underestimated.

In summary, our results suggest that aepEX may be used to evaluate the degree of sedation at anesthetic induction of midazolam, thiopental and ketamine with propofol.

## Conclusion

Bispectral index values remained high at the time of LOR, pre- and post-intubation when ketamine was used for anesthetic induction with propofol, showing dissociation with the clinical level of consciousness. AepEX values, on the other hand, decreased appropriately at the time of LOR, pre- and post-intubation in all groups, and aepEX may be adequately represented the depth of sedation. These results suggest that aepEX might be more efficacious than BIS as an indicator of hypnosis during the anesthetic induction of midazolam, thiopental and ketamine with propofol.
